# The “symbiosis” between high-quality register data and value-based health care and the role of local, national, and international registers in the development and promotion of value-based healthcare projects

**DOI:** 10.1186/s12913-026-15593-w

**Published:** 2026-09-15

**Authors:** Peter Cnudde, Ola Rolfson, Louisa Huxtable-Thomas

**Affiliations:** 1 Department of Orthopaedics, Hywel Dda University Healthboard, Prince Philip Hospital, Bryngwynmawr, Llanelli, SA14 8QF UK; 2 Value-based Health and Care Academy, School of Management, Swansea University, Bay Campus, Swansea, SA1 8EN UK; 3 Registercentrum, Swedish Arthroplasty Register, Gothenburg, VG 41345 Sweden; 4 Department of Orthopaedics, Institute of Clinical Sciences, Sahlgrenska Academy, University of Gothenburg, Medicinaregatan 3, Gothenburg, 41390 Sweden

**Keywords:** Value-based health care, Registers, Evidence-based medicine, Quality improvement, Pathways

## Abstract

**Background:**

Since 2006, the idea of Value-Based Health Care (VHBC) has gained traction, introducing the need for value from a patient’s view as well as a health system’s view. Arthroplasty registers and their international body, the international society of arthroplasty registries (ISAR) seek to improve outcomes for individuals receiving joint replacements; at face value, register data forms part of a VBHC approach. This study explored the symbiotic relationship between register data and VBHC and contributions between registers and evidence-based medicine (EBM), quality improvement and pathways.

**Methods:**

The study used exploratory, semi-structured interview methods with a sample of regionally, nationally, and internationally recognised register experts. Fifteen participants were provided with some contextual questions prior to the interview. Interviews were held using online technology, and transcriptions stored and analysed using NVivo software. The thematic analysis was performed using qualitative coding to seek trends in experts’ views on registers with a confirmatory coding and analysis undertaken by a social science researcher.

**Results:**

Improved patient outcomes and linking value to care by contributing to EBM as part of the function of the registers were supported by interviewees and interviews revealed the importance of clinician-led registers in the integration of outcome measures and the implementation of an evidence-based practice. By providing the four stakeholders (the patient, the clinical teams, the health system, and the society) with information to improve decision-making protocols, support clinical decisions on effective implant choices and pre- and post-operative care regimes, registers have contributed to improved outcomes. The role of the registers in supporting the development of arthroplasty pathways was found to be more ambiguous and variable across territories and between practitioners. Whilst the focus of the registers has been on improving outcomes, cost-savings for the healthcare systems were considered a bonus.

**Conclusions:**

The analysis supports a link between the principles of VBHC including the aim to reduce unwarranted variations, and the evidence-generating potential of arthroplasty registers, outlining a common purpose in improving outcomes and value for patients, surgical teams, healthcare organisations and society. Further research is planned to refine views and provide further insights in the complete pathway and potential symbiosis.

**Supplementary Information:**

The online version contains supplementary material available at https://doi.org/10.1186/s12913-026-15593-w.

## Background

Codman, considered the father of registries, used his meticulously collected data collection to assess his outcomes and improve a strategic plan for future patients [1–3]. Registers (defined as an organised system of collecting outcome data at population level) have been a source of information for patients, clinical teams, healthcare institutions, medical device providers and organisers/payors of healthcare [4]. Local and national registers have been associated with improving outcomes, improving safety, adoption of best practice, and reducing cost [5, 6]. Within orthopaedics and especially joint replacement surgery, national, regional, and institutional registers and their international society, the international society of arthroplasty registries (ISAR) have been driving clinical improvements [7–12]. Arthroplasties registries have been at the forefront of register research for patients besides monitoring implant performance. Through the provision of feedback to the different stakeholders, based on high quality prospectively collected data, the outcomes of joint replacement surgeries and the care delivered have been improving over the years [7–12].

Since 2006, Value-Based Health Care (VBHC), defined as a healthcare delivery concept designed to maximise patient health outcomes relative to the costs incurred per patient, has gained traction. VBHC emphasised the need for value from a patient’s perspective as well as health systems, with a focus on measuring and improving outcomes that matter to the patient [13–15]. Within orthopaedics and specifically the provision of arthroplasty, clinical pathways, outcome measurement and VBHC have become a commonly used concept over the last decades [16–19]. Clinical pathways are defined as the patient’s journey from the time of diagnosis until long-term follow-up, involving complex interventions, mutual decision-making and multiple interactions and interdependent intervention components [16–19]. Integrated care pathways are the backbone of VBHC [20–22].

The concept of VBHC requires healthcare delivery systems to reassess value, moving beyond financial outcome and instead towards less tangible outcomes that matter to patients, including quality of life, achieving personal goals and even societal value. Organisations like the International Consortium for Healthcare Outcomes Measurement (ICHOM) and ISAR could potentially play a role in enabling the intangible value to the patient to be quantified. This can potentially be achieved through defining what matters most to patients or being able to measure trends in outcomes. In order to measure and improve outcomes, there is a need to define, measure, record, and analyse the relevant data and, on face value, register data forms part of the VBHC approach. As a result, defining and agreeing a standard set of outcome measures has been on the agenda of the different registers, ISAR and ICHOM [23–25].

In order to assess the congruence in their mission statements, the aim of this research project was to evaluate the potential for symbiosis (defined in a non-biological context, as a mutual beneficial, dependent relationship between people, businesses or organisations) between high-quality register data within the field of arthroplasty and VBHC, exploring the contributions of the registers to Evidence-Based Medicine (EBM), quality improvement and the value creation potential of the registers in the process. Furthermore, we also explored the role of registers in developing pathways, the measurement of cost, potential links with electronic health records and concerns about Patient-Reported Outcome Measures (PROMs). To summarise, this project wanted to explore the following questions:

1. How have local, national, and international arthroplasty registers contributed to the development of EBM and quality improvement?
2. How have local, national, and international arthroplasty registers contributed to the development of patient-centred arthroplasty pathways?
3. And finally, if there was a symbiotic relationship between high-quality register data and the underlying ideas of VBHC?

## Methods

This qualitative study used exploratory, semi-structured interview methods with a sample of regionally, nationally, and internationally recognised register experts and followed the guidance of the Evidence-Based Medicine Working Group on qualitative research, the Consolidated Criteria for Reporting Qualitative Research (COREQ), and the CORR guidance on importance, robustness and support for better and efficient care [26–28].

### Research team and reflexivity

#### Personal characteristics

The interviews were conducted by the first author (PC), a male orthopaedic surgeon with 25 years of experience caring for patients requiring joint replacement surgery. He has extensive experience working with clinical registries and has conducted registry-based quantitative research using data from an established registry. In 2018, he earned a PhD in Clinical Sciences from the University of Gothenburg [29]. The research team (PC, OR, and LHT) has extensive expertise in both qualitative and quantitative research methodologies.

#### Relationship with participants

The participants, invited to take part in the research project, were all approached prior to the start of the project during conferences and were provided with an invitation email (supplementary document 1), explaining the reasons for and aims of doing the research as well as the background of the researcher. The first author had previously worked with 2 participants in a clinical and academic setting.

### Study design

#### Theoretical framework

This study is an in-depth inquiry, understanding the dynamics of registers and the associations quality improvement, EBM, pathways and VBHC, and is underpinned by the case study approach [30, 31] utilising reflexive thematic analysis for inductive theory elaboration [32].

#### Participant selection

Participants in this study were required to answer questions relating to arthroplasty registers that required a level of expertise and experience. As such a purposive sample was sought from orthopaedic surgeons with register expertise, members and non-members of ISAR. Where respondents to this request did not participate, an element of snowball sampling approach was utilised to seek further possible replacements (see Table 1). In total, fifteen registry experts participated in the study, having been provided with contextual questions prior to the interview and consented to the interview and the recording of the interview (supplementary document 2). As shown in Table 1, six of the 21 people approached refused and/or passed the request on to a colleague on the basis of retirement or time constraints. The interviewees, of which the majority were practising orthopaedic surgeons (eleven), were or had been actively involved in registers and register research and were from different countries and continents. Of the other four, one interviewee was a non-medical register director, another interviewee was involved in outcome measurement research, and two interviewees were orthopaedic surgeons who retired from surgical practice. Regional and national registers were both represented by the participants, some of which had roles on both regional and national registers and were able to provide answers relating to both (see Table 1). Some of the interviewees had been involved from the start of their respective organisation whilst others had been involved with their registers more recently. There was a combination of interviewees from both mature and developing registers, from bigger and smaller countries/regions (first essential aspect) [26].

**Table 1:** Participants for interview with location of interviewee, contribution to regional or national register and whether agree to participate (Y = yes, N = no)

By Interviewee	Continent	Regional	National	Agreed and interviewed
1	Europe	N	Y	Y
2	Europe/ North America	Y	Y	Y
3	Europe	N	Y	Y
4	Europe	N	Y	Y
5	Europe	N	Y	Retired/N
6	Europe	N	Y	Y
7	Europe	N	Y	Y
8	North America	N	Y	Forwarded to colleague/N
9	North America	N	Y	Y
10	North America	N	Y	Forwarded to colleague/N
11	North America	N	Y	Forwarded to colleague/N
12	Asia-Pacific	N	Y	Y
13	Asia-Pacific	N	Y	Y
14	North America	N	Y	Y
15	North America	Y	N	Y
16	Europe	Y	Y	Y
17	Europe	Y	Y	Y
18	Europe	N	Y	No reply/N
19	North America	Y	N	Y
20	North America	Y	N	Forwarded to colleague/N
21	Europe/ North America/Worldwide	N	Y	Y

**Table 2:** 13 generated themes with summaries and relevant quotes

Theme	Summary	examples/quotes
1. Evidence-Based Medicine (EBM)	• Registers have a significant role to play and are contributing to EBM and the evidence, generated from register-sourced EBM can be translated into guidelines and pathways within a learning healthcare environment. • The effect is bidirectional: leading the change but also generating hypotheses.	*“It is very exciting that there is a significant link between EBM and the registry.” (Asia-Pacific surgeon)* *“The registers introduce a rigor to the testing of changes in clinical practice in the real world*,* which is really where EBM needs to go*,* because it so much faster.” (international*,* non-surgeon)*
2. Pathways	• With regards to pathway development, there is a variation in answers, but smaller registers (Kaiser Permanente, MARCQI, Geneva) are more actively involved in developing pathways, whereas other registers are providing the data to support the development of pathways or are using the register to confirm if pathways changes are working and changes in practice, based on register results, are having the desired effect. • Extremely limited data on complete journey and usually limited to the time of the operation and some follow-up. • Although checking the complete pathway could be possible in Sweden and maybe MARCQI. • In the Netherlands there is a project to align outcome form the registers into pathways and some non-arthroplasty registers were built around the pathway to enable the development of practice guidelines and clinical care standards were the basis of the register to enable measurement.	*“Most of our clinical pathways and what we have developed*,* have been in partnership with other organisations across the country.” (North America*,* surgeon)* *“The clinical pathways are actually necessary for the registration*,* because you have to fit the registration in the clinical pathway.” (Europe*,* non-surgeon)* *“Pathways have undoubtedly been developed because of the contribution by the register*,* but we recognised early that our job really was to get the information out there.” (Europe*,* surgeon)*
3. Quality improvement (QI)	• QI has been the main focus of the registers, leading to improved care. • The reduction in mortality and revision rate can be attributed to the role of the registers. • The importance of the feedback loop and performing full circles of QI. The role of the procedure outcome analysis in QI has been recognised as one of the strengths.	*“There are three questions for QI: are you any good? Are you getting better? And how do you know? You need systems such as registries to leverage the data.” (Europe*,* surgeon)* *“Our stated goal is to make… the best place in the world to have a joint replacement.”* *(North America*,* surgeon)*
4. Power of data	• The registers are considered to be a very powerful platform to analyse outcomes and compare. • The opportunities are vast, the use of dashboards might improve the data accessibility and programs such as ‘patients like me’ can aid shared decision-making and patient decision tools the unlocking of some opaque doors to improve population health and disease management. • There are questions whether the volume of data starts opening the door for causal inference.	*“The data*,* we have helped accumulate*,* has had a massive influence on how we work with best practice guidelines*,* more day-case activity*,* recognising risk factors*,* screening*,* how we select patients*,* how we optimise patients for surgery*,* and how we may use implants differently for different indications*,* all based on the gathered evidence.”* *(Europe*,* surgeon)*
5. Patient-reported Outcome Measures (PROMs)	• Whilst PROMs are a longstanding part of some registers, some have only recent got PROMs as an essential requirement. • Still a low capture rate in some registers and now getting aligned with national requirements in some countries.	*“The part we are missing is the PROMs portion and a more robust fashion and a more robust reporting of that.”* *(North America*,* surgeon)*
6. Patient satisfaction	• Patient satisfaction could potentially be an excellent measure and could demonstrate trajectories, aid in decision-making and could be picked up early after discharge.	*“One of the questions is how satisfied are you with your joint replacement as it does a good job encapsulating the patients’ expectations*,* their improvement in pain and function and their complication rate.” (North America*,* surgeon)*
7. Value and Value-Based HealthCare (VBHC)	• The value-creation function from the registers is certainly recognised. Whilst there are some concerns of VBHC being too broad and maybe a hype, data sourced from registers has been used in implementing VBHC and creating value through collecting data, analysing data, and feeding back that data to the stakeholders. • Programs such as “patients like me,” and “Choosing wisely” add the extra dimension that the patients will benefit from. • The high-quality real-world data, focussing on accessibility, appropriateness, effectiveness, and safety will cover the various aspects of value generation. • Looking at the end-result using standardised outcome measures (in cooperation with International Consortium for Health Outcome Measurements) will improve the outcomes of the interventions and will contribute to a better population health.	*“I found it (i.e. the data within the registers) to be really the data that Michael Porter had talked about*,* but that is not readily available*,* it wasn’t basically available anywhere in the world. This proves what the Swedes have done with the registries*,* proves that our idea… can be done in practice.” (international*,* non-surgeon)* *“The real value of the registers from a VBHC principle is that it represents unbiased real-world representative data”* *(Europe*,* non-surgeon)*
8. Costs	• Cost, whilst not recorded in any of the registers, could be an additional very valid recorded variable, there would be concerns of who is accountable and what cost would have to be recorded.	*“Registries provide already a lot of value; they can do even better*,* and they can collaborate with the cost side.”* *(North America*,* surgeon)*
9. Efficiency	• Some evidence of developments based on the work of the registers to improve efficiency.	*“You don’t have to wait too long to see what they are sort of doing now in terms of maintaining efficiency and quality outcomes.” (Europe*,* surgeon)*
10. Way forward	• An agreement on minimal datasets, timing and how to measure outcomes would improve transparency and aid the comparability between different registers. • High level independent statistical advisors, knowledge sharing and using technical platforms using standardised pre, peri and postoperative notes would be extremely helpful. • The International Prosthesis Library (IPL) and international outlier analysis are notable examples of international collaboration and a future vision. • There is a need to speak the same language, attempting to catch the complete cycle and providing feedback to Clinical Practice Guidelines (CPG’s) will improve the care for patients.	*“Outcomes should be harmonized” (Europe*,* surgeon)* *“It would take an enormous amount of time and buy-in and it would need alignment consensus meetings.” (Europe*,* surgeon)* *“I would like to see ISAR getting everybody on the same page.” (Europe and North America*,* surgeons AND Europe*,* non-surgeon)* *“One of the key things is just to standardize their measures.” (international*,* non-surgeon)*
11. Electronic Health Record (EHR)	• There are opportunities for the EHR to be integrated with the recordings of complications, recording of PROMs and are pathway compliant. • Potentially Artificial Intelligence (AI) would be able to subtract information from the EHR and transfer it to be used in registers, avoiding duplication.	*“My hope is that we really put some effort behind looking at technological advances for digital data capture*,* through NLP or potential AI algorithms.” (Europe*,* non-surgeon)*
12. Benefits for the healthcare sector	• There are numerous identified direct benefits for the Healthcare sector with net financial savings, avoidance of poorer performing implants (early warning system), less complications and service transformations, based on data from registers.	*“We have to do work to sort of justify or existence to the payer and that work has shown that our projects have provided significant improved value.”* *(North America*,* surgeon)*
13. Risks and concerns	• Data quality and safety are ongoing concerns.	*“There was a suspicion about what we are doing and who gets and controls the data*,* but that concern has been settled now.” (Europe*,* surgeon)*

#### Setting

Interviews were conducted by the researcher (PC) and held between August 2022 and November 2023 using virtual meetings (researcher and single participant) and the description of the sample can be found in Table 1.

#### Data collection

Individuals were contacted and invited by email, explaining the background, aims of the study, and an attached document with anchoring questions as well as the themes for the semi-structured interview (supplementary document 2). Following agreeing informed consent, interviews, using exploratory, semi-structured interview methods, were organised at a time convenient for the participant. Interviews were held virtually using Teams^®^ and transcribed using digital technology (Teams^®^) with secondary manual checks for accuracy and to redact identifying data. No repeat interviews were carried out. Transcripts were not returned to participants for comment or correction. Researcher’s notes and reflections were collected in a lab book during the interview and the second listening, and corrected transcriptions were stored as word documents. After two initial pilot interviews, the questions were discussed within the research team and adjusted to retain the focus on the research questions. This final list of prompts is included in supplementary document 2. Interviews lasted an hour on average.

As well as the sample providing assurances of diversity (mature versus younger, different continents, different sizes), depth and nuances (2 participants from same register) interviews were sought from respondents matching the sampling criteria until no new insights, trends or codes were generated from the new interviews. A figure of between 9 and 17 interviews or 12 interviews has been quoted to empirically assess saturation [33, 34]. This initially occurred at 12 interviews and a further three were held to confirm this.

### Data analysis

NVivo^®^ software (Lumivero, CO, USA) was used to store, code, and further analyse the transcripts (second and third essential aspect) [26]. A 6-step approach as described by Braun and Clarke was used to perform a reflexive thematic analysis by following the familiarisation phase and identifying key themes initially (phase 1), generating initial keywords and codes (phase 2) [35, 36]. Using a combination of inductive and deductive methods utilising a combination of preconceived themes linked to the interview questions, followed by a phase of inductive supplementary theme identification from iterative interrogation of the transcript data themes were generated (phase 3) and reviewed in phase 4 (fourth essential aspect) [26, 35, 36]. These themes, reviewed in phase 4 were subsequently defined and named in phase 5, prior to the write up (phase 6) [35, 36]. A second qualitative researcher from a non-cognate field, business, (LHT) reviewed the interview data and confirmed agreement the coding and themes as well as the point of saturation. The three researchers (PC, OR, LHT) grouped the themes to correspond to the research questions and additional information. Participants were invited to provide feedback on the findings following the presentation at the ISAR Hamburg 2024 meeting.

## Results

The six-phase process was performed in a logical sequential, however non-linear order, utilising an iterative process to question and confirm coding.

Watching, reviewing notes, transcribing, and reading the interviews, enabled familiarisation, highlighting initial findings and interesting or unexpected phrases or key words. The researcher noted practical examples of where difference in outcomes was achieved such as implant choice, the use of cement, infection prevention and blood clot (DVT/PE) prevention.

15 initial high-level, codes were identified: power of data, quality improvement, pathway(s), value, patient satisfaction, EBM, cost, way forward, concern, risks, Electronic Health Records (EHR), PROMs, efficiency, benefit to healthcare stakeholders and who maintains/owns the register. The 15 initial high-level codes were interpreted and combined into themes for the next phase of which 13 generated themes, which underwent confirmatory analysis, and are summarised within Table 2.

When matched to the three research questions, answers were generated as follows:

1. How have local, national, and international arthroplasty registers contributed to the development of EBM and quality improvement?
  - Registers have a significant role in contributing to EBM by both generating the evidence but also generating hypothesis that need further evidence (theme 1).
  - QI has been the focus of the registers and procedure outcome analysis is one of the big strengths. Registers have been proven to benefit the HC sector with net savings, avoidance of poorer performing implants and there is evidence of service transformation using register-based data (themes 3, 9, 12).
  - Registers are considered to be a powerful source of data, but the opportunities to use the data quicker and better should be explored and the volume of data could start opening the doors to prove causal inference (theme 4).
2. How have local, national, and international arthroplasty registers contributed to the development of patient-centred arthroplasty pathways?
  - The role of registers in the development of pathways is a bit more ambiguous and variable, from completely hands-off to a direct involvement and even aligning the outcomes that are measured within the register around the pathway. The input is mainly focussed on the time of the operation and is certainly not as would be considered an integrated care pathway. In all cases register data has enabled pathway development (theme 2).
  - The collection of PROMs is not generalised, there is still a concern about low capture rates. The role of measuring patient satisfaction as one of the standard outcome measures needs to be studied (themes 5, 6, 10).
3. And finally, if there was a symbiotic relationship between high-quality register data and the underlying ideas of VBHC?
  - The value-creation function from the registers is certainly recognised. Data from registers has been used in implementing VBHC and creating value through collecting data, analysing data, and feeding back that data to the stakeholders. Programs such as “patients like me,” and “Choosing well” add the extra dimension that the patients will benefit from. The high-quality real-world data, focussing on accessibility, appropriateness, effectiveness, and safety will cover the distinct aspects of value generation. Looking at the end-result using standardised outcome measures (in cooperation with ICHOM) will improve the outcomes of the interventions and will contribute to a better population health (theme 7, 9).
  - The role of the register in costs needs to be studied (theme 8, 10).
  - The role of an accepted EHR and the linkage with registers opens opportunities (theme 11).

Additional themes were identified which provided further detail on the practical utilisation of registry data, relevant to the implementation of VBHC:

Standardisation, speaking the same language, the use of technical platforms, sharing knowledge and the use of independent statistical advisors should be embraced (theme 9,10).

Data quality and confidentiality are remaining concerns and risks (theme 13).

Un-themed:

A single participant noted a further suggestion that a register could potentially also be built around a pathway with the practice guidelines and clinical standards forming the basis of what performance or which quality indicators need to be measured and in what timeframe to help assessing how each hospital (and or surgeon) is performing against that ideal care standard or model of care (minority view).

## Discussion

Arthroplasty registers have been a valued source of information for orthopaedic surgeons for the last five decades and the interest in their publications is increasing [7, 37, 38]. Whilst on face value, outcome data (from the registers) in the form of CROMs (Clinician-Reported Outcome Measures) and PROMs (Patient-Reported Outcome Measures) form part of the VBHC approach to deliver healthcare systems that patients value, this has never been established [39–41]. Hence this study was commenced to evaluate a potential symbiosis between high-quality register data and VBHC using qualitative research methods.

The respondents agreed that registers, and the output from registers, were actively contributing to improving outcomes and as such contributing to quality improvement. The use of registry data, knowledge, and infrastructure can further support outcome measurement, necessary to assess the value of the care and the interventions and support diverse quality improvement projects, through both data and feedback. Registers are contributing to EBM by generating evidence but also by generating hypotheses that in turn require research [7]. Signal detection and identifying outliers early have contributed significantly to the outcomes of arthroplasty surgery and this was also supported in the literature [10, 42, 43].

Integrated care pathways are the backbone of VBHC [20–22]. The role of the registers in supporting the development of arthroplasty pathways was found to be more ambiguous and variable. The input from the registers, however, was limited to the timeframe around the operation (perioperative) and certainly did not cover the time, prior to the surgery (preoperative) and only to a limited extent the time after the surgery (postoperative) as would be suggested by the VBHC philosophy [13, 15, 44, 45]. There was evidence from a single register that was aligning the outcomes, measured within the register, around the pathway. Measuring the outcomes to make judgements on whether value has been achieved is an important aspect of VBHC. Whereas the CROMs were collected and analysed with a great degree of coverage and completeness across the territories of the study participants, the collection of PROMs was still not generalised and there were concerns about low capture rates in many of the registers. The additional value of patient satisfaction (as an additional outcome) has been suggested [46, 47].

Based on the thematic analysis, particularly examining the link between the principles of VBHC and the evidence-generating potential of arthroplasty registers, a common purpose in improving outcomes and value for patients, surgical teams, healthcare organisations and society could be supported. Data from registers has been used in implementing VBHC, improving outcomes, and creating value through collecting, analysing, and feeding back that data to the stakeholders [4, 16, 48]. Programs such as “patients like me” and “Choosing wisely” add an extra dimension that patients will benefit from [49–51]. While joint replacement surgery is considered one of the most rewarding surgical interventions for patients and the population [52]. Reviewing the data in light of non-financial values, yields the following conclusions: The technical value of Registries within arthroplasty is well established as it relates to the optimisation of outcomes to achieve the best possible outcomes with the available resources and practically this involves techniques/actions that contribute to minimising complications and reducing length of stay but also benchmarking of implants. Personal value, i.e. those outcomes that matter to the patients, is interpreted as the impact that correct use of PROMs (and CROMs) as recorded in in the registries has on learning about and improving the effectiveness of interventions on managing pain, improving physical function, mobility and quality of life. The allocative value relates to the impact that Registry data, collected at population level, can have on enabling well-being of the population and the equitable and fair contribution of healthcare resources in that region. The societal value of joint replacement surgery is well established as it allows the patient to return to work, remain independently and engage socially – but only in as much as those outcomes are enabled. Registries allow that assessment to be made with certainty [53].

Within the registries the recording, analysis and feedback of key measures, relating to outcome (such as PROMs and CROMs), but also the study of the effect on implant choice, surgeon and hospital characteristics contribute to measuring the different value pillars and is based on real-world data. Cost analysis, deeper integration with linking with other data sources such as imaging, EHR (spanning primary and secondary care plus medication registries), and socio-economic datasets and finally improved analytical methods will allow for real-time monitoring and predictive modelling further enhancing the different value pillars [53]. The high-quality real-world data, focussing on accessibility, appropriateness, effectiveness, and safety covers some of the different aspects of personal, technical, allocative and societal value generation [54]. Looking at the end-result using standardised outcome measures was seen to improve the outcomes and to contribute to better population health. The role of the registers in understanding costs would need further investigation. While the prime focus of the registers has been on improving outcomes, cost savings have been considered a bonus for the society and are a key aspects of the Value assessment made in VBHC, and as a result are currently being investigated [4].

The participants in this study were convinced of the strengths of registers in contributing to EBM. Registries were considered to generate observations which could lead to new hypotheses as well as test the available best evidence from Randomised Control Trials (RCT’s) in real-world scenarios and had a bidirectional effect, hence the increase in publications based on register data [37]. Quality improvement projects, signal, and outlier detection were felt to provide benefits for patients, surgeons, healthcare systems and society. A further mention was made by a single participant. who identified that future registers should or could be more aligned to outcome measures. They described this as a way to record and feed back in alignment with the clinical care standards and the identified and agreed performance indicators based on the clinical practice guidelines for the intervention or diagnosis. While not a commonly noted code, this did imply that existing registers were not viewed as sufficiently aligned at this time.

Interviewees identified some common opportunities during their interviews. These included using register-nested RCT’s with more frequency, to use linkage to improve access to missing variables, but also improving data quality and opportunities to prove causation [9, 55–59]. Measuring outcomes and efficiency of the interventions along the complete pathway as well as cost was suggested as a way to provide additional value to the registers [60, 61]. Using dashboards to provide quicker feedback and help with the shared decision-making process prior to surgery was considered a good potential use of the data collected through registers. International cooperation was strongly recommended by the majority of interviewees, and clear conditions for this were put forward, such as agreed minimum data sets, data sharing opportunities (within accepted frameworks), the use of independent statistical advisors, standardisation, inclusive of using the same terminology, technical platforms, and using standardised reporting in order to improve comparability were agreed as a potential to improve the working of registers individually as well as internationally.

### Limitations

The patient should be considered the main stakeholder in arthroplasty surgery [52, 62]. There was no patient involvement in the planning of the research or as participant. Unfortunately, the dual challenges of limited time and resources prohibited patient involvement across all territories of the study for this initial exploration of the link between VBHC and registries. There is however patient involvement within the different registers. For the interviews, only orthopaedic surgeons and non-medical colleagues involved in the working of the registers were interviewed, increasing a potential risk of bias. A potential selection bias of interviewees, whilst possible, was minimised by inviting participants from different geographical areas, surgeons and non-surgeons, associations with regional, national, or institutional registers as well as different maturity of the registers. Arthroplasty registers have been at the forefront of register research for patients with implantable devices but also in other non-implant related branches of the medical field. Arthroplasty registers have been leading the way in the development of other registers. And whilst the disease-specific PROMs might not be generalisable, other outcome measures (both PROMs/ CROMs/…) will be. Registers and the outputs of the registers (inclusive the generation of research) are considered mainstream in the countries with a register tradition, but register-based research has known limitations such as data completeness, reporting bias, variation in data collection practices, and differences across healthcare systems, but these limitations are outside the scope of this research project.

The participants remained wary of the risks of poor data quality, the potential problem of GIGO (= Garbage in, Garbage out) and the data confidentiality [63]. One of the concerns when performing qualitative research is data saturation and based on the results of the analysis, and in line with previous published guidance, data saturation was believed to be achieved [33, 34]. The main stakeholders, discussed within the research project were limited.

to patients, healthcare providers (surgical teams and hospital) and the society, there are however other stakeholders such as the industry, regulatory agencies amongst others [64]. Finally, researchers are always at risk of bias in the research process and whilst the experiences, assumptions, ideas, and personal interest have initiated, sustained and hopefully enhanced the research in a reflexive way, all efforts have been made to avoid directing the research in a predetermined way [65, 66].

## Conclusion

The evidence from this study confirms that registers, their overarching societies and VBHC have a common purpose in improving the outcomes for patients undergoing joint replacement surgery. As such, this study provides the link between data, evidence and VBHC concept and confirms that the mission statements of ISAR and ICHOM are well aligned with the VBHC concept and are contributing to the successful symbiosis to improve outcomes for individual patients. Future quantitative and evaluative research should include the return on investment and how registers create value and how the complete pathway of joint replacements could be analysed.

### Ethical approval

## Supplementary Information

Below is the link to the electronic supplementary material.

Supplementary Material 1

Supplementary Material 2

## Data Availability

The datasets used and/or analysed during the current study are available from the corresponding author on reasonable request.

## Abbreviations

AI: artificial intelligence
CROMs: Clinician-Reported Outcome Measures
EBM: Evidence-Based Medicine
HER: Electronic Health Record
FU: follow up
GIGO: Garbage In, Garbage Out
HC: Healthcare
KP: Kaiser Permanente
ICHOM: International Consortium for Healthcare Outcomes Measurement
IPL: International Prosthesis Library
ISAR: International Society of Arthroplasty Registries
NLP: Neuro-linguistic programming
PROMs: Patient-Reported Outcome Measures
QI: Quality Improvement
RCT: Randomised Controlled Trial
VBHC: Value-Based Health Care
