# Supplementary material for: The “symbiosis” between high-quality register data and value-based health care and the role of local, national, and international registers in the development and promotion of value-based healthcare projects
Source: BMC Health Serv Res. 2026 Sep 15;26:1465. doi: 10.1186/s12913-026-15593-w (PMC13695288; doi:10.1186/s12913-026-15593-w)
Supplement: Supplementary file 1 [file 12913_2026_15593_MOESM1_ESM.docx]

Dear colleague,

I hope this email find you well.

As a colleague, who has been involved in the development and running of arthroplasty registers, I would like to ask you if you would be willing to give up some of your valuable time to participate in my project, called: The “symbiosis” between high-quality register data and value-based health care (VBHC). The role of local, national and international registers in the development and promotion of value-based healthcare projects.

The project is part of an ISAR-funded fellowship and will analyse your views on the role of registers in VBHC. The need for relevant and good quality outcome data to guide decisions and policies and measure and compare outcomes is paramount. And hence, I do believe there is a big role for the registers and the register societies to fully engage with the multi-disciplinary members of value-based healthcare teams to constructively cooperate on the how, when and which data has to be collected in a prospective manner in order to guide future decisions, healthcare analysis and implementation of future policies. Your views and experience on the current and future role of the registers and the interactions between arthroplasty registers and VBHC would be very welcome. Some preliminary findings, based on pilot interviews have already been presented at the ISAR Montreal meeting. I am hopeful to present the findings at the next ISAR meeting in Hamburg 2024.

The project is supervised by Assoc Prof. Louisa Huxtable-Thomas, School of Management, Swansea University, Wales, UK and Prof. Ola Rolfson, Swedish Arthroplasty Register and Department of Orthopaedics, Gothenburg University, Gothenburg, Sweden. The project has been approved by the School of Management’s Research Ethical Committee. The study will involve an interview at a time of your convenience. It is anticipated that the interview will take about one hour to complete and there will be a list of short context questions forwarded to you to complete prior to the interview. The interview questions will also be shared with you and all the participants prior to the interview. Participation is completely voluntary, and you will be able to withdraw from the study at any time. There is no funding available to take part in the interview.

Every effort will be made to keep participants anonymous in the final published study, and the analysis method will aggregate findings. However, due to the limited number of participants and in an environment where participants are familiar with each other’s work, it will be difficult to provide full anonymity

Please find enclosed a consent form, some short context questions as well as the subject questions in preparation for the interview.

Looking forward to receiving a positive answer and speaking to you in the near future,

Kind regards,

Peter

Peter Cnudde, M.D., PgCert (Clin. Leadership), PhD

Consultant Orthopaedic Surgeon Hywel Dda University Health Board, Llanelli, Wales, U.K.

D.B.A. student, School of Management, Swansea University, Wales, U.K.

Enc.

1. Consent form
2. Short context questions and interview questions
