# Supplementary material for: The “symbiosis” between high-quality register data and value-based health care and the role of local, national, and international registers in the development and promotion of value-based healthcare projects
Source: BMC Health Serv Res. 2026 Sep 15;26:1465. doi: 10.1186/s12913-026-15593-w (PMC13695288; doi:10.1186/s12913-026-15593-w)
Supplement: Supplementary file 2 [file 12913_2026_15593_MOESM2_ESM.docx]

**The “symbiosis” between high-quality register data and value-based health care.**

**The role of local, national and international registers in the development and promotion of value-based healthcare projects.**

**Research Questions**

**Pre interview context questions (anchoring)**

1. Your role and your register
   a. Provide a brief history (or reference to history) of your register
   b. Describe your role within the register
   c. Describe your professional background
   d. Describe who owns/governs your register
2. Quality improvement projects within your register

Tell me briefly about a couple (max 2) of big achievements and how it has impacted care for the patients?

3. Are you familiar with the VBHC principles of Michael Porter?

Yes/No

**Interview questions**

4. Has your register been involved in opportunities in supporting the development of clinical pathways?
   a. Example
   b. Outcome
   c. Effect for
      1. Patient
      2. Surgical care teams
      3. Organisation
      4. Society
   d. Concerns
5. Has your register been involved in opportunities in supporting the development of Evidence-Based Medicine/Practice?
   a. Example
   b. Outcome
   c. Effect for
      1. Patient
      2. Surgical care teams
      3. Organisation
      4. Society
   d. Concerns
6. Has your register been involved in opportunities in supporting the development of VBHC principles or changes?
   a. Example
   b. Outcome
   c. Effect for
      1. Patient
      2. Surgical care teams
      3. Organisation
      4. Society
   d. Concerns
7. More generally how can local, national and international registers aid the development and promotion of value-based healthcare projects?
8. How do you believe the principles of VBHC get integrated in the work of registers?
   a. Good example
   b. Strengths
   c. Weakness
   d. Opportunities
9. How can local, national and international registers aid the development patient-centred arthroplasty pathways based on Evidence-Based Practice (EBP) and Value-Based Health Care principles?

Thank you
